# Prostate Cancer: genetics in practice now and in the future

**DOI:** 10.1186/s13053-025-00310-1

**Published:** 2025-03-25

**Authors:** Jana McHugh, Elizabeth Bancroft, Zsofia Kote-Jarai, Rosalind Eeles

**Affiliations:** 1 The Institute of Cancer Research, 123 Old Brompton Road, London, SW7 3RP UK; 2 The Royal Marsden NHS Foundation Trust, Fulham Road, London, SW3 6JJ UK

## Abstract

Prostate Cancer (PrCa) is one of the most common cancers worldwide and causes a significant healthcare burden. Recent predictions estimate the incidence of new cases of PrCa will double from 1.4 million in 2020 to 2.9 million by 2040.

The known risk factors for PrCa are increasing age, family history, ancestry and genetics. PrCa is one of the most heritable of the more common cancers. The heritability of PrCa is due to both rare moderate to high-risk monogenic variants and more common variants known as single nucleotide polymorphisms (SNPs) which can be used to calculate a polygenic risk score (PRS) for PrCa, while there is some of the genetic risk as yet unexplained. In recent years more PrCa risk-associated SNPs have been identified, increasing over time with the inclusion of more persons of diverse ancestry in studies. The identification of germline variants known to be associated with increased PrCa risk and disease aggressiveness has led to targeted treatments for certain pathogenic variant carriers.

This is a mini review of how the genetics of PrCa can impact on screening and early detection of the disease and the treatment and management of the disease when diagnosed.

## Background

PrCa is the most common cancer in men in 112 countries worldwide [1]. Recent predictions estimate the incidence of new cases of PrCa will increase annually from 1.4 million in 2020 to 2.9 million by 2040 [2].

Despite how common PrCa is and its high burden of morbidity and mortality globally, currently there is no agreed population screening programme worldwide unlike other common cancers like breast, colorectal or cervical cancer. As we have entered the era of precision medicine with more and more genetic data available to us, we can look at utilising these data to enable early diagnosis and also assist in targeting effective precision treatments to those affected by the disease.

PrCa has a wide spectrum of clinical aggressiveness, and this makes balancing overdiagnosis of indolent disease and early recognition of lethal PrCa challenging. In the past proposed screening programmes have relied on prostate specific antigen (PSA) testing. Although decreased PrCa specific mortality has been seen as the data mature [3] with ~ 30% reduction in PrCa mortality in persons screened using PSA testing compared with non-organised opportunistic PSA testing, this comes at the price of overdiagnosis. The large US based Prostate Lung Colorectal Ovarian (PLCO) study [4] and the European Randomised Study of Screening for Prostate Cancer (ERSPC) [5] when initially reported led to concerns about overdiagnosis of indolent PrCa and overtreatment. The CAP (Cluster Randomised Trial of PSA testing for Prostate Cancer) trial based on a single PSA screening test failed to show an association between screening and PrCa mortality [6] though as data has matured a modest improvement was shown with screening [7]. These data have meant most countries have not adopted PSA-based PrCa screening at population level. In recent years clinicians have tended to adopt opportunistic PSA testing in the absence of guidelines, which has made study design challenging when trying to find the optimal PrCa screening algorithm, and currently there is no agreed consensus on population level screening for PrCa worldwide. Risk-stratification has been suggested an optimal screening pathway for PrCa and incorporating a polygenic risk score may be helpful in this in combination with other risk factors like age, ancestry and family history.

## Heritability of Prostate Cancer

PrCa is known to have one of the highest heritability of the more common cancers at 58% [8]. The genetic predisposition to PrCa is made up of 1) common variants in the population that confer a modest impact on PrCa risk and 2) rarer monogenic variants that have a modest increase in lifetime risk of developing PrCa for individuals carrying a variant. The most well-known genes in which pathogenic variants increase risk of PrCa include *BRCA2, BRCA1, MSH2, MSH6, MLH1, HOXB13, ATM, PALB2, NBN* and *CHEK2* [9]. Monogenic variants are identified using DNA sequencing methods, while SNPs have been identified through genome wide association studies (GWAS), and to date 451 PrCa risk-associated SNPs have been identified [10]. These PrCa risk-associated SNPs may confer lower risk individually but multiplicatively can add to up to at least 2.7-fold increased risk of PrCa for those in the top decile of PRS [11]. This may now be estimated as even higher given the more recent identification of 451 PrCa risk associated SNP [10], while the previous estimate of 2.7-fold was estimated when less than half of these had been identified.

**Fig. 1:**
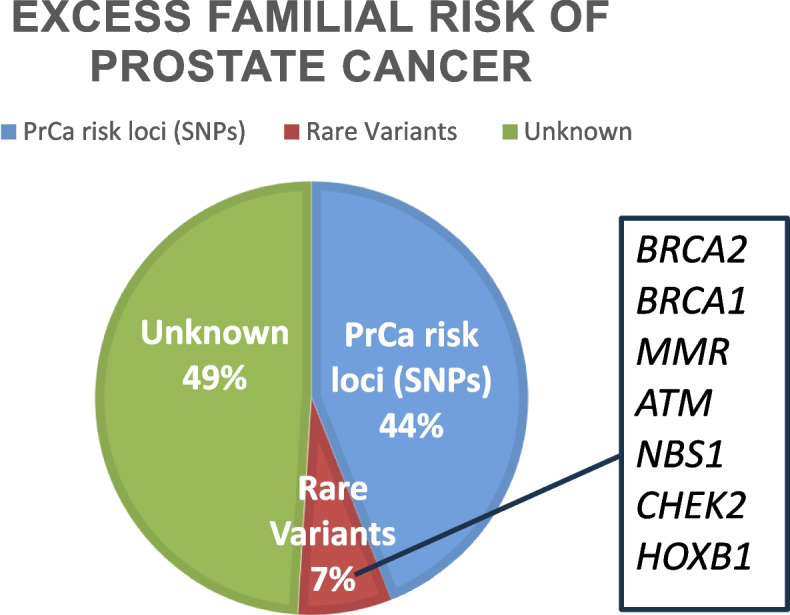
Excess familial risk in prostate cancer causes

In Fig. 1 above, the excess familial risk of PrCa causes is outline with GWAS identified SNPs currently estimated to account for approximately 44%. Approximately 49% is as yet unexplained. Rare variants in certain genes associated with hereditary cancer syndromes like *BRCA2* and *BRCA1, MMR* = mis-match repair genes such as *MSH2* and *MSH6* associated with Lynch Syndrome, *ATM, NBS1* and *CHEK2* among others and *HOXB13* is PrCa specific account for approximately 7%.

The National Comprehensive Cancer Network (NCCN) guidelines recommend the following genes for germline testing currently include *BRCA2, BRCA1, ATM, PALB2 and CHEK2* and mismatch repair (MMR) genes; *MSH2, MSH6, MLH1, PMS2* and *HOXB13* [12]. In the United Kingdom (UK) there are 8 of the above genes listed for testing on the National Genomic Testing Directory and *NBN* and *HOXB13* are also considered PrCa predisposition genes and may be included in future clinical guidelines [13].

Germline testing is useful for: 1) diagnosis and screening potential, 2) prognosis; considering more aggressive management for variant carriers with localised PrCa and considering different targeted treatment options for those with advanced PrCa, 3) informing risk for family members; allows for cascade testing and counselling leading to personalised surveillance.

European guidelines from the European Society of Medical Oncology (ESMO) now recommend germline testing for those with PrCa [14]; echoing the USA NCCN guidelines. ESMO guidelines recommend germline testing for DNA damage repair (DDR) genes associated with cancer predisposition syndromes in patients with a family history of cancer and should be considered in all patients with metastatic prostate cancer. Tumour testing should be considered for homologous recombination genes and mismatch repair defects or microsatellite instability in patients with metastatic castrate resistant PrCa [14]. A recent ESMO update has recommended germline follow-up of tumour-only sequencing including defining the seven ‘most-actionable’ cancer susceptibility genes (*BRCA1, BRCA2, PALB2, MLH1, MSH2, MSH6, RET*) in which germline follow up is recommended regardless of primary cancer type [15].

Traditionally, germline testing would take place in clinical genetics units and involved two clinic visits: an initial testing and education visit and then another visit to discuss results. More recently technologies such as video/phone-based clinical counselling are routinely used to help to meet increasing demand for testing [16].

The 2019 Philadelphia Prostate Cancer Consensus Conference suggests optimal genetic consent should include: discussion of the purpose of testing and the types of possible results (pathogenic/likely pathogenic variants, benign/likely benign variants or variants or unknown significance, no variants detected), the possibility of identifying hereditary cancer syndromes or other cancer risks, the importance of cascade family testing, legal issues; and the US Genetic Information Non-discrimination Act (GINA) law [9].

Mainstream testing has been adopted as standard practice in breast, ovarian and colorectal cancer in many countries and this is now being implemented for PrCa. There is a need to streamline genetic testing as we use it more routinely and ideally make it accessible to all and feasible for oncology teams to deliver.

## Implications of germline testing results

### Localised PrCa

Studies have shown that *BRCA* variant carriers, in particular *BRCA2,* develop disease at younger ages and have a higher risk of developing metastatic disease and poorer survival [17–19]. NCCN guidelines recommend considering DNA repair variant status when discussing active surveillance [12]. Germline variants in *BRCA2, BRCA1* and *ATM* are associated with a higher likelihood of reclassification/upgrading among those undergoing active surveillance as a management strategy for the PrCa [20]. Based on these data, carriers of mutations/variants should be closely monitored and could potentially benefit from earlier definitive treatment approaches.

There are early data to suggest that *BRCA* mutation/variant carriers versus non carriers have poorer outcomes with external beam irradiation but no difference with prostatectomy [18].

### Advanced PrCa

In advanced PrCa patients with DNA repair gene mutations/variants have a higher response rate to targeted agents. In 2020 the Food and Drug Administration (FDA) approved two new targeted agents: poly-adenosine diphosphate-ribose polymerase (PARP) inhibitors: Olaparib and Rucaparib for those with germline or somatic DNA damage repair gene mutations [21].

Early data suggest that platinum chemotherapy is also effective in prostate tumours with DNA repair deficiency [22–24].

## Implications of genetics on PrCa screening

The increasing incidence of PrCa means it is vital that early detection be prioritised. With the advent of improved imaging with multi-parametric magnetic resonance imaging (mpMRI) [25, 26] adding imaging to PSA and other biomarkers may be the best approach for PrCa screening, helping to reduce the number of unnecessary biopsies. The ideal screening programme would be able to identify those at risk of developing the disease and prioritise these for more intensive screening. The development of polygenic risk scores for diseases has been continuing as GWAS data continue to be accrued and evaluated. Creating a PrCa PRS could be useful in risk-stratifying in population screening for PrCa [27]. More PrCa risk loci continue to be identified as GWAS report on populations of diverse ancestries reflecting improvements in inclusion and diversity in genetic research [28].

To date, there are 451 SNPs identified that confer PrCa risk, the most recent additions were found using a large multi-ancestry dataset [10]. Using these to develop provide an inclusive and ancestry diverse PRS will offer a method of stratifying PrCa risk in populations. This can help to guide timing and frequency of PrCa screening. As outlined, there is currently no globally accepted screening strategy for PrCa despite its incidence worldwide. There are ongoing studies looking at the clinical utility of integrating genetic profiling into PrCa screening algorithms.

## Conclusions

PrCa is highly heritable and using genetics can help to target screening to those at the highest risk of developing PrCa. Utilising a PRS can help to risk stratify the population for PrCa population screening programmes. There are growing treatment choices for those with PrCa who carry high-risk variants so germline testing for those meeting family history criteria and those with metastatic disease can help to guide personalised precision medicine in this common cancer.

## Data Availability

No datasets were generated or analysed during the current study.
